# Deep Cutaneous Candidiasis With Costal Osteomyelitis Following Pectoralis Major Myocutaneous Flap Reconstruction: A Case Report

**DOI:** 10.7759/cureus.78210

**Published:** 2025-01-29

**Authors:** Hiroyuki Goda, Koh-ichi Nakashiro, Satoshi Hino, Nobuyuki Kuribayashi, Daisuke Uchida

**Affiliations:** 1 Department of Oral and Maxillofacial Surgery, Ehime University Graduate School of Medicine, Toon, JPN

**Keywords:** adenoid cystic cancer, costal osteomyelitis, deep cutaneous candidiasis, oral cancers, pectoralis major myocutaneous flap reconstruction

## Abstract

*Candida* is a yeast commonly found in various environments. It usually coexists with the skin without causing harm. It may exhibit pathogenicity when local or systemic immune defense mechanisms are compromised, which creates conditions favorable for its proliferation. This report discusses a case of invasive cutaneous candidiasis with rib osteomyelitis that developed at the donor site of a pectoralis major myocutaneous flap. The patient was a 58-year-old man who underwent reconstruction with a pectoralis major myocutaneous flap for adenoid cystic carcinoma in the midline floor of the mouth. Six months postoperatively, a reddish mass appeared at the donor site in the anterior chest. *C. albicans* was isolated from this lesion. Despite undergoing curettage and receiving oral antifungal treatment, the lesion showed no improvement. The reason for the treatment failure remains unclear. Consequently, surgical resection, including the fourth rib, was performed. Based on clinical, histopathological, and microbiological evaluations, the patient was diagnosed with deep-seated cutaneous candidiasis with rib osteomyelitis. The patient has been followed for 19 years postoperatively, with no evidence of recurrence of the primary floor-of-mouth carcinoma or candidiasis.

## Introduction

Deep-seated cutaneous candidiasis is a condition caused by *Candida* species, which form ulcers and abscesses in the dermis and subcutaneous tissue. *Candida* species are among the most common causative pathogens of nosocomial infections [1], with over 150 species identified to date. Among these, *C. albicans *is the most frequently reported causative agent of deep-seated cutaneous candidiasis. This disease was previously considered a rare opportunistic infection. However, in recent years, the incidence and severity of this condition have increased, primarily due to risk factors such as the widespread use of broad-spectrum antibiotics, antineoplastic agents, and immunosuppressive drugs. Its etiology is multifactorial, involving disruption of normal microbial flora and impaired immune function [2]. This report presents a case of invasive cutaneous candidiasis with rib osteomyelitis that developed at the donor site of a pectoralis major myocutaneous flap used for reconstructing a surgical defect following resection of an oral malignancy.

## Case presentation

A 58-year-old male presented to our institution in November 2005 with a chief complaint of swelling in the floor of the mouth. The case was reviewed in accordance with ethical standards, and appropriate permissions were obtained from the patient, including informed consent for the use of clinical data. His past medical and family history were unremarkable. The initial clinical diagnosis was a median ranula. In February 2006, the mass located in the midline of the floor of the mouth was resected under general anesthesia. Histopathological examination of the resected lesion revealed adenoid cystic carcinoma, and additional surgical intervention was deemed necessary. In March 2006, the patient underwent a series of surgical procedures, including a tracheostomy, bilateral neck dissection, resection of the malignant tumor in the floor of the mouth, and reconstruction using a pectoralis major myocutaneous flap. The pectoralis major flap, measuring 60 × 40 mm, was harvested from the left chest. The donor site was closed with buried sutures using 3-0 Vicryl (Ethicon, Inc., Bridgewater, USA) and skin sutures using 4-0 nylon. Postoperative wound healing was uneventful, and the patient was discharged in June 2006 without additional treatment. No recurrence or metastasis was observed during the six-month postoperative follow-up period.

**Figure 1:**
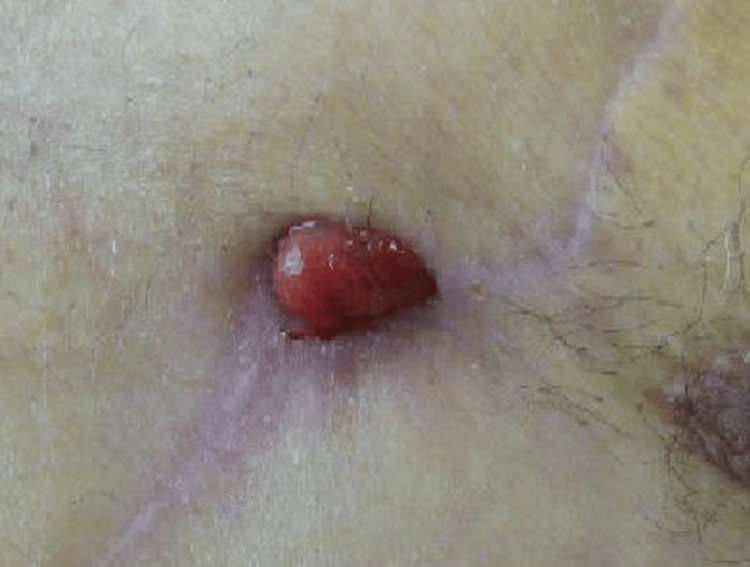
An initial anterior chest photograph A granulomatous lesion measuring 17 × 12 mm with localized erythema and tenderness was observed at the site of the pectoralis major flap harvest.

In September 2006, the patient presented with a painless mass, measuring 17 × 12 mm, at the donor site of the left pectoralis major flap (Figure 1).

**Figure 2:**
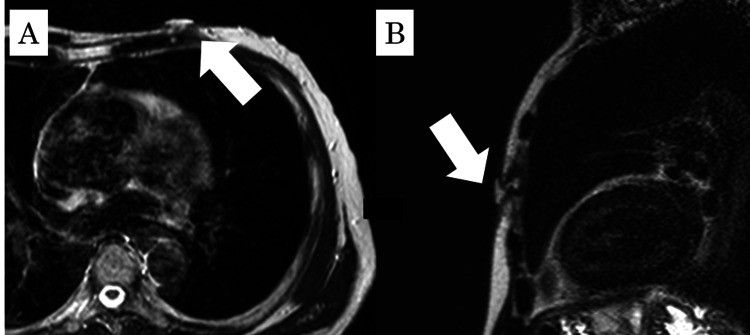
MRI images (T2-weighted sequence) (A) Axial view; (B) Sagittal view. A 20-mm lesion with high signal intensity was observed in the left anterior chest (arrows).

Blood tests, including hematological and biochemical examinations, showed no abnormalities. Locally, a well-demarcated mass with redness and fluctuation was observed at the donor site. There was no evidence of surrounding induration. Curettage was performed under local anesthesia, and histopathological examination revealed inflammatory granulation tissue predominantly composed of neutrophils. Microbiological tests identified *C. albicans*. In October 2006, a recurrence of the lesion was observed, and oral fluconazole at a dose of 100 mg per day was administered for 10 days. However, no improvement was noted. Subsequently, additional curettage was performed by the dermatology department, and the patient was treated with oral terbinafine hydrochloride at 125 mg per day for 28 days. Despite these treatments, the lesion recurred, accompanied by redness and tenderness. Further evaluation in November 2006 included an MRI, which revealed a 20-mm mass in the left anterior chest. The lesion was continuous with the fourth rib and costal cartilage (Figures 2A, 2B).

**Figure 3:**
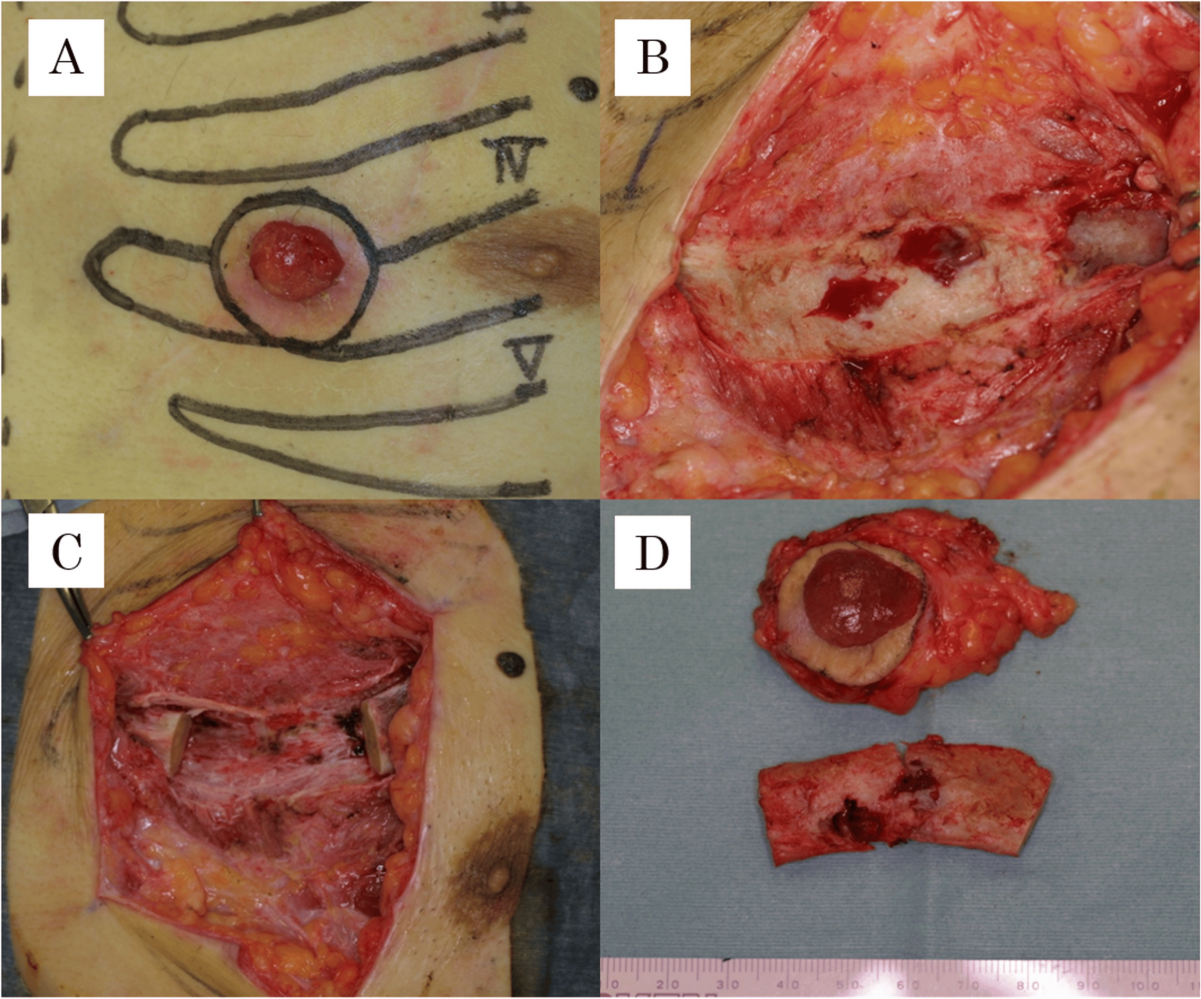
Intraoperative photographs (A) Preoperative design; (B) After tumor resection: two areas of penetrating bone destruction were observed in the fourth costal cartilage; (C) After segmental resection of the fourth costal cartilage; (D) Resected specimen

Additionally, serum Candida antigen was positive at 2+. Surgical intervention was performed under general anesthesia by the plastic surgery team. The procedure included resection of the fourth rib and costal cartilage as well as debridement of necrotic tissue in the left chest (Figures 3A-3D).

Following the resection, the wound was closed with dermal and skin sutures. Postoperatively, cefazolin sodium at 2 g per day was administered intravenously for three days. The postoperative course was uneventful, and the patient was discharged on the 13th postoperative day. Over a 19-year follow-up period, no recurrence of the lesion has been observed.

**Figure 4:**
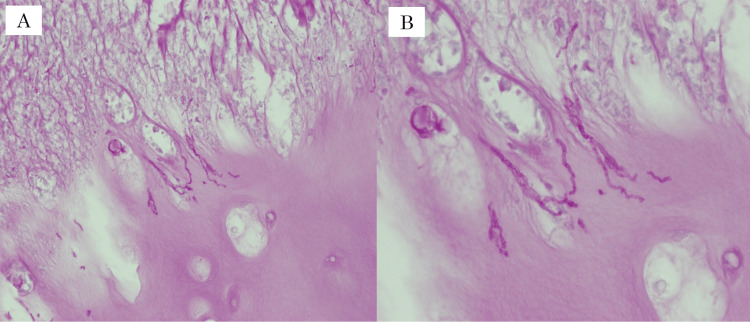
Histopathological images of the resected specimen (A) Periodic acid-Schiff (PAS) staining (×200); (B) PAS staining (×400). PAS staining revealed PAS-positive fungal hyphae within the degenerated costal cartilage.

Histopathological examination of the resected specimens revealed granulation tissue involving necrotic tissue and costal cartilage. The granulation tissue extended from the skin surface to the cartilage and was accompanied by scarring. The central portion of the costal cartilage exhibited cavitation and necrotic degeneration. Periodic acid-Schiff (PAS)-positive fungal hyphae were detected, leading to a final histopathological diagnosis of fungal granuloma (Figure 4).

## Discussion

Deep-seated cutaneous candidiasis is an opportunistic infection that is relatively uncommon in clinical practice and occurs in patients with compromised immune defense mechanisms. This disease has a high mortality rate, ranging from 40% to 50% [3], with approximately 50% of cases arising from hematogenous dissemination [4]. On the other hand, advancements in cancer therapy and organ transplantation in recent years have led to increased susceptibility to immunosuppression, resulting in a higher risk of deep-seated fungal infections [5,6]. In this case, the patient had a history of malignancy but lacked typical risk factors such as advanced age, diabetes mellitus, and use of anticancer drugs, steroids, or immunosuppressants. The patient also had a good nutritional status. To the best of our knowledge, no previous reports have documented deep-seated cutaneous candidiasis originating from the donor site of a pectoralis major myocutaneous flap used for oral reconstruction.

Deep-seated cutaneous candidiasis, also referred to as fungal granuloma in some cases, can be classified into endogenous and exogenous infections [7-10]. Endogenous infections occur when *Candida* species, which are commensals in the gastrointestinal tract and skin, acquire pathogenicity in immunocompromised hosts. Exogenous infections, on the other hand, result from *Candida* entering through surgical wounds or medical devices. In the present case, the lesion coincided with the surgical wound, and MRI findings revealed a relatively well-defined lesion within the rib. This suggested that the infection originated from the surgical site, with partial wound dehiscence at the suture site possibly serving as a predisposing factor.

The pathogenesis of *Candida* infections involves several key steps. Initially, *Candida* adheres to host cells through adhesins such as Als3 and Hwp1 [11,12]. Following adhesion, *Candida* undergoes morphological changes, transitioning from a yeast form to a hyphal form, enhancing its invasive potential [11]. The hyphae then penetrate deep tissues through physical extension and enzymatic actions, including those mediated by secreted aspartyl proteases (SAPs) [8]. Additionally, *Candida* forms biofilms, which contribute to treatment resistance and immune evasion [13]. Hypha-specific toxins, such as candidalysin, damage host cell membranes and further facilitate tissue invasion [11].

The recommended treatment for deep-seated cutaneous candidiasis includes systemic antifungal agents, with echinocandins, a class of antifungal drugs, as the first-line therapy. This class includes caspofungin, micafungin, and anidulafungin, while fluconazole and liposomal amphotericin B serve as alternative treatment options [4,14]. The treatment duration is typically at least two weeks following the disappearance of *Candida* from the bloodstream or until the resolution of lesions, which may require several months. In this case, *Candida* was identified within the cartilage matrix of the rib, where antifungal drug penetration was insufficient. Consequently, surgical resection of the affected area was necessary.

## Conclusions

Deep-seated cutaneous candidiasis has a high mortality rate and is associated with risk factors such as malignancy and immunosuppressive therapy. Lesions presenting as indurations, abscesses, or ulcers at surgical sites or catheter insertion points should raise suspicion for this disease. While the pectoralis major myocutaneous flap remains a valuable reconstructive method, its use requires careful consideration of the potential risks of deep-seated cutaneous candidiasis. This case underscores the importance of meticulous surgical site management and prompt intervention for early signs of abnormality to reduce postoperative risks of invasive infections. Granulomatous lesions at the site of surgical scars should prompt further evaluation for infection and malignant recurrence. In addition, early intervention, such as biopsy and appropriate antimicrobial therapy, may facilitate timely diagnosis and improve patient outcomes.
